# The combined effects of lactoperoxidase system and whey protein coating on microbial, chemical, textural, and sensory quality of shrimp (*Penaeus merguiensis*) during cold storage (4 ± 1°C)

**DOI:** 10.1002/fsn3.669

**Published:** 2018-06-04

**Authors:** Maryam Farshidi, Mohammad Yousefi, Ali Ehsani

**Affiliations:** ^1^ Faculty of Nutrition and Food Sciences Department of Food Science and Technology Tabriz University of Medical Sciences Tabriz Iran

**Keywords:** antimicrobial coating, lactoperoxidase, shelf life, shrimp, whey protein

## Abstract

Growth and reproduction of gram‐negative bacteria has a pivotal role in spoilage of seafood products. In order to identify the effect of lactoperoxidase system (LPOS), an antimicrobial activity was added to whey protein solution at the levels of 0 (control group), 1.25%, 2.5%, 5%, and 7.5%. Then, the shrimp samples were coated with immersion method and stored in the refrigerator for 16 days. In this period, the microbial tests of *psychrotrophic bacteria, mesophilic bacteria, Pseudomonas fluorescens, Pseudomonas* spp., and *Shewanella putrefaciens*, chemical tests of total volatile basic nitrogen, thiobarbituric acid, and pH, and sensory evaluation were carried out on the days of 0, 4, 8, 12, and 16. Adding LPOS in the coating resulted in a decrease in the total specific spoilage organisms and TVB‐N. There was no significant effect for the TBA test. The levels of LPOS showed a positive correlation with the acceptable measurement in the sensory evaluation.

## 1 INTRODUCTION

The enzymatic and microbial activities of fresh shrimp have caused it to be more perishable than many other foods. The effect of the microbial activity is usually the dominant agent limiting the shelf life and safety of shrimp during refrigeration storage and transportation. During the past several decades, certain methods have been developed in order to increase the shelf life of shrimp in an attempt to prevent the consumer health risks (Al‐Dagal & Bazaraa, 1999). Furthermore, traditional methods such as cook‐chill processes and storage were commonly used to avoid the corruption of fresh shrimp. Different natural antimicrobial and chemical compounds, antioxidants, polymers, and biotechnology products have been developed as well and effectively used in foodstuff industry. In addition, natural herbal extracts, particularly essential oils and bacteriocins such as nisin and LPOS have been found as appropriate additives for the conservation of products (Lu, Ding, Ye, & Liu, 2010). Using edible coatings has expanded the shelf life of shrimp (Nowzari, Shábanpour, & Ojagh, 2013). In this procedure, a thin layer of edible and biocompatible coating is used to protect the shrimp from the physical and chemical damage and delay the microbial growth and increase the safety. Moreover, the additional benefits of the method are reducing waste packaging and producing new food (Valverde, Valero, Martínez‐Romero, Guillén, Castillo, & Serrano, 2005).

The growing consumer worries about the human health impacts of chemical preservatives have encouraged several studies to focus on the edible coatings containing natural antioxidant and antimicrobial properties. Chitosan, Chitin, and their derivatives have been utilized for years in the processing of marine products due to their antibacterial and antioxidant activities (Kamil, Jeon, & Shahidi, 2002). Furthermore, shellfish produced ink of sepia has been found to retard the corruption of shrimps as a reliable edible coating (Sadok, Abdelmoulah, & El Abed, 2004).

Bacterial contamination is one of the reasons of spoilage of seafood products. Recent researches have proved that usually in special refrigeration conditions, the corruption occurs mainly due to the presence of specific microorganisms that are identified and introduced as organisms involved in corruption. According to the studies of researchers, some species of gram‐negative organisms, particularly *Pseudomonas fluorescens* and *Shewanella putrefacience,* have been introduced as the organisms involved in the corruption of most fish, mollusks, and crustaceans during the storage at cold temperatures (ice and refrigerator) (Xu, Lin, Sui, & Cao, 2012).

The main purpose of adding the antimicrobial components for packaging films is enhancing the antimicrobial effect which leads to control the surface corruption growth agents and pathogenic (Kuorwel, Cran, Sonneveld, Miltz, & Bigger, 2011; Ouattara, Simard, Piette, Bégin, & Holley, 2000). Moreover, instead of mixing antimicrobial components directly with the food, combining them into coating solutions lets the functional impact on the food surface be localized. So the use of such films in foods such as meat is very practical. The advantage of this method is a slow and gradual release of antimicrobial compounds into the food material (Coma, Sebti, Pardon, Deschamps, & Pichavant, 2001).

Lactoperoxidase (LPO) enzyme, a glycoprotein in milk, saliva, and tears of mammals is introduced as one of the most important enzymes used in food industries as a biological antimicrobial agent with broad spectrum which can be utilized in food packaging. Investigations have shown that this enzyme has bactericidal impact on gram‐negative bacteria along with inhibitory impact on gram‐positive bacteria. In addition, antifungal and antiviral activity of this enzyme has been reported (Seifu, Buys, & Donkin, 2005; Yener, Korel, & Yemenicioğlu, 2009). LPOS has made of these three compounds: lactoperoxidase, thiocyanate (SCN−), and hydrogen peroxide (H_2_O_2_) where the enzyme catalyzes oxidation of SCN− by H_2_O_2_ and to produce antimicrobial compounds such as hypothiocyanite (OSCN−) and hypothiocyanous acid (HOSCN) That this substances are having the potential inhibition of microorganisms through oxidation of sulfhydryl (SH−) groups in their enzymes and proteins systems. These substances have the potential inhibition of bacteria through oxidation of sulfhydryl (SH−) groups in their enzymes and protein systems (Cissé, Montet, Tapia, Loiseau, & Ducamp‐Collin, 2012). The cytoplasmic microorganism membrane damage caused by the oxidation of sulfhydryl (SH−) groups has been reported as the most important principle of the destruction of microbial cells (Mohamed, Clementine, Didier, Gérard, & Noëlle, 2013; Min, Harris, & Krochta, 2005).

Whey protein is the good source for biodegradable edible coating and is suggested in food industry. By adding plasticizer, clear, bland, and flexible edible films based on water will be produced which have excellent oxygen, oil, and aroma barrier features (Gennadios, 2002). According to Min, Harris, and Krochta (2005), LPOS did not significantly change the tensile features, oxygen permeability, and whey protein films color. Accordingly, the aim of this study was to develop a solution of whey protein as a protective antimicrobial coating or low‐cost food by blending, LPOS as an antimicrobial enzyme system with both the bactericidal and bacteriostatic impacts to enhance antimicrobial quality, mainly on particular corruption of bacteria and extend shelf life fillets of shrimp in the refrigerator.

## 2 MATERIALS AND METHODS

### 2.1 Materials

Lactoperoxidase system was composed of glucose oxidase (GO; Sigma‐Aldrich), LPO (150 U/mg, Sigma‐Aldrich), D‐(α)‐glucose (Glu, Sigma‐Aldrich), H2O2 (Merck, Germany), and potassium thiocyanate (KSCN, Bioserae, France). Glycerol, a plasticizer for improving coating flexibility, was obtained from Merck (Frankfurt, Germany). Whey protein (80% protein) was purchased from DMV Co. (Veghel, the Netherlands).

### 2.2 Preparation of LPOS

Lactoperoxidase system preparation was performed as described by Cissé et al. (2012). The ratios of the LPOS components based on weight were 0.35, 1.00, 108.70, 2.17, and 1.09 for GO, LPO, Glu, H_2_O_2_, and KSCN, respectively. To prepare LPOS, the compounds were separately dissolved in 50 ml of phosphate buffer (pH 6.2, Sigma‐Aldrich); and then, 15.5 mg lactoperoxidase enzyme was added to it. To increase the antibacterial activity of LPOS, the final solution was incubated at 23°C under shaking at 160 rpm for 24 hr (Min, Krochta, & Rumsey, 2007).

### 2.3 Shrimp fillet preparation

Shrimps used for experiments were obtained from the Persian Gulf (Iran). They were gutted, beheaded, washed with tap water, and then transferred immediately to the laboratory on ice.

### 2.4 Preparation of the whey protein solution followed by treatment of shrimp fillets

Mixing 10 g Whey Protein with 100 ml distilled water and stirring in a Controlled temperature of 90°C to result in a clear mixture prepared whey protein solution (WPS). Glycerol, as a plasticizer, was added to the solution (Min et al., 2005). LPOS concentrations prepared at 1.25%, 2.5%, 5%, and 7.5% (v/v) were added to the WPS. In order to determine the optimum LPOS concentrations in WPS, a preliminary disk diameter test was carried out with the concentrations of 0.31%, 0.63%, 1.25%, 2.5%, 5%, 7.5%, and 10% LPOS (v/v) in a solution of whey protein. The bacterial suspension was adjusted to 1 × 107 colony‐forming Units (CFU)/ml and spread on the iron agar LYNGBY with the help of sterile cotton swab. After that, 6 mm in diameter filter paper disks were impregnated with 20 μl of the each solution and placed on the surface of insemination. After incubation at 4°C for 2 hr, the plates were placed in an incubator at 30°C for 3–4 days. The antibacterial strength was investigated by measurement of the diameter of inhibitory zone. It was found that the concentration of LPOS <1.25%v/v produced no specific growth inhibition zone of spoilage bacteria, while the concentration of 10% had extremely high viscosity (Shokri, Ehsani, & Jasour, 2015). So, LPOS concentration in the ranges of 1.25% to 7.5% was selected in this study.

Fillets samples were selected randomly according to six coating formulations that were randomized by following treatment:

(1) Coating formulation (control): 0% LPOS‐ 0% WPS. (2) 1.25% LPOS‐WPS. (3) 2.5% LPOS‐WPS. (4) 5% LPOS‐WPS. (5) 7.5% LPOS‐WPS.

For each treatment, approximately 20 shrimp fillets were dipped in coating solutions of well mixed for 1 min. The ratio of fillets immersed in the solution was 1:2 (Erkan, 2013). After immersion, fillets were removed and permitted to be drained and then put in polyethylene bags and stored at 4 ± 1°C for 16 days.

### 2.5 Bacteriological analysis

Ten grams sample of shrimp meat was aseptically mixed with 90 ml of 0.1% peptone water with stomacher (Pulsifier^®^, UK) and was homogenized for one minute. In all cases, serial dilutions were prepared in peptone water 1.0% of the shrimp homogenized solution. All bacteria were counted by pour plate method in the appropriate media. Viable *mesophilic* bacteria were cultured using Nutrient Agar (Merck, Darmstadt, Germany) after incubation at 37°C for 48 hr. *Psychrotrophic* bacteria were cultured using King Agar (Merck, Darmstadt, Germany) by incubating at 21°C for 48 hr. H2S producing bacteria, including *Pseudomonas fluorescens* as white colonies and *S. putrefaciens* as black colonies, were cultured using Iron Agar LYNGBY (Laboratorios Conda, Madrid, Spain) after incubation at 30°C for 3–4 days. For the *Pseudomonas* spp. count, cetrimide agar (Merck, Darmstadt, Germany) was incubated at 37°C for 48 hr. All counts were mentioned as log10 CFU/g.

### 2.6 Chemical analysis

#### 2.6.1 Determination of pH value

Five grams of fillet shrimp was homogenized in 25 ml of distilled water for 30S, and the solution pH was determined using pH meter (pH 510; Eutech^®^ CyberScan, Singapore) at room temperature according to AOAC (2005).

#### 2.6.2 Measurement of thiobarbituric acid reactive substances (TBARS)

Thiobarbituric acid (TBA) test was done based on the method proposed by Benjakul and Bauer (2001). One gram of meat was homogenized for 2 min with 9 ml of 0.25 molequi/L HCl solution containing 15 g/100 ml trichloroacetic acid (TCA) and 0.375 g/100 ml 2‐thiobarbituric acid (TBA). The mixture was heated in a boiling water bath for 10 min, then immediately cooled with running water. After that, the mixture was centrifuged at ‘3,500’ rpm for 15–20 min. Then the absorbance was measured at a 532 nm in front of the blank solution consisting of 5 ml of TBA reagent. TBARS values can be expressed as mg of malonaldehyde (MA)/kg of sample.

#### 2.6.3 Measurement of total volatile basic nitrogen

Method of Goulas and Kontaminas (2005) was used to measure total volatile basic nitrogen (TVB‐N) value. Ten grams of minced meat was mixed with 2 g of MgO and 250 ml of distilled water, and one drop of silicon was added as antifoam and then moved to a flask. After that, this blend was distilled into a receiver flask containing 20 ml of a 3% aqueous solution of boric acid with an indicator (0.1 g of methyl red and 0.1 g of methylene blue in 100 ml of ethanol). Then, the distillate was titrated with 0.01 N HCl. The TVB‐N value has been calculated by a volume consumed of the HCl and was expressed as mg/100 g shrimp of meat.

### 2.7 Sensory analysis

The rating tables provided by Erkan (2012) were used to describe the sensory evaluation of fish meat. For this reason, 20 semitrained individuals were recruited. Sensory evaluation included color, odor, texture, and overall acceptability with the scoring of 1 to 9. According to the scoring tables, the ranges of 1–3.9, 4–6.9 and 7–9 were assigned as unacceptability, moderate acceptability, and high acceptability, respectively.

### 2.8 Statistical analysis

Results were expressed as mean ± standard deviation. All results were analyzed using SPSS software (version 18.0 for Windows, SPSS, Inc., Chicago, IL, USA) and were performed to one‐way analysis of variance (ANOVA) to find the effects of experimental conditions. Accordingly, the Shapiro–Wilk’s test and the homogeneity of variance were used to examine the normality of data determined by Levene’s test. Data were followed by Duncan’s post hoc test. The statistically significant differences were set at *p* < .05.

## 3 RESULTS AND DISCUSSION

### 3.1 Determination of bacteriological changes

The results of the microbial analysis showed that the accompanying of LPOS with the coating material of whey protein decreased the growth of microbes in shrimp muscles during 16 days of being kept in the refrigerator. This decrease was more salient in higher levels of LPOS presence. According to Min et al. (2005), lactoperoxidase system (LPOS) that exists along with GO causes continuous production of oxidized material which has antibacterial effects. Also, this study has confirmed the possibility of continuous production of oxide materials with antibacterial activity in the presence of LPO and GO system in whey protein coating that the shrimp fillet samples were coated with.

**Figure 1:**
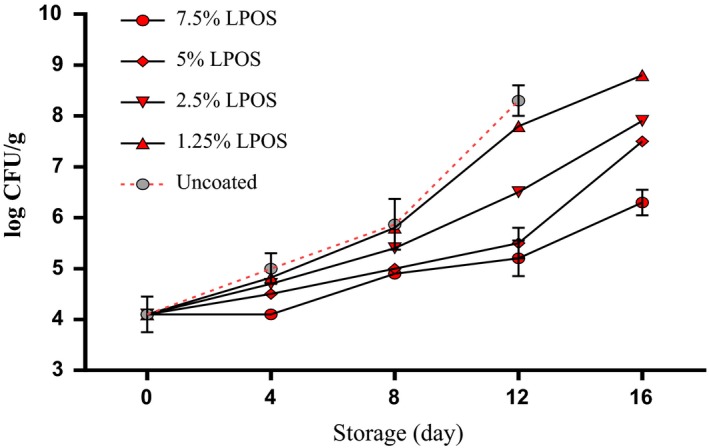
The *mesophilic* bacteria changes of shrimp fillets as affected by LPOS‐WPS during refrigerated storage (4 ± 1°C). Values are the mean ± *SD*. LPOS, lactoperoxidase system; WPS, whey protein solution

Significant statistical difference (*p* < .05) in count *mesophilic aerobic* bacteria was observed significantly between the groups treated with different levels of LPOS and the control group (Figure 1). On the other hand, At the Level of 7/5% LPOS‐treated samples, less *mesophilic* bacteria were counted in comparison with other groups. So at the end of holding period, the number of aerobic *mesophilic* bacteria was less than 7 log cfu/g which is less than the number of bacteria that can be considered as an indicator of seafood spoilage (Gram & Huss, 1996). As well as our findings, similar results were reported about incorporated efficacy of LPOS and edible coating solution against *mesophilic aerobic* bacterial growth in chicken and fish (Jasour, Ehsani, Mehryar, & Naghibi, 2015; Molayi, Ehsani, & Yousefi, 2018; Shokri, Ehsani, & Jasour, 2015; Yousefi, Farshidi, & Ehsani, 2018).

**Figure 2:**
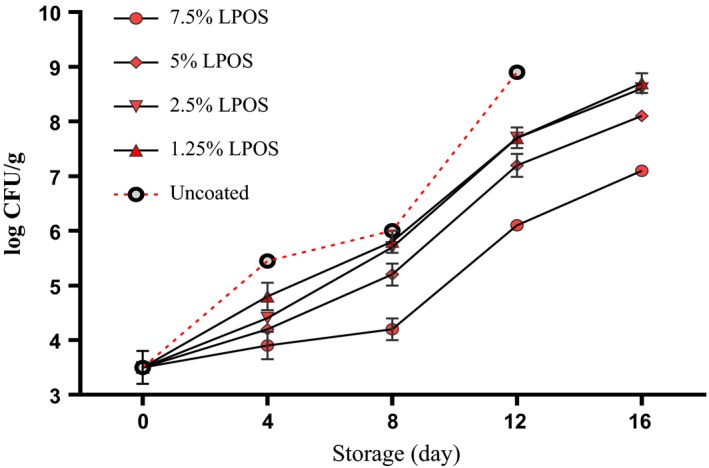
The *psychrotrophic* bacteria changes of shrimp fillets as affected by LPOS‐WPS during refrigerated storage (4 ± 1°C). Values are the mean ± *SD*. LPOS, lactoperoxidase system; WPS, whey protein solution

The *psychrotrophic bacteria* had a major impact on shrimp spoilage during cold storage (Sallam, Ahmed, Elgazzar, & Eldaly, 2007). There was a significant difference in counting them between LPOS‐treated group and control group. By increasing the concentration of LPOS in coating solutions of whey protein, fewer bacteria were observed and counted (Figure 2). The fewest counted bacteria were related to two upper levels of LPOS (5, 7.5%) although the difference between these two levels was insignificant at the end of the tests. However, at the end of the storage in the refrigerator (Day 16), the *psychrotrophic* bacteria numbers were more than 7 log CFU/g for each of the groups studied. This result is in accordance with Rostami, Abbaszadeh, and Shokri (2017) who reported that combined effects of LPOS‐whey protein coating and vacuum packaging on extension of the shelf life of Pike‐Perch fillets during cold storage (4°C).

**Figure 3:**
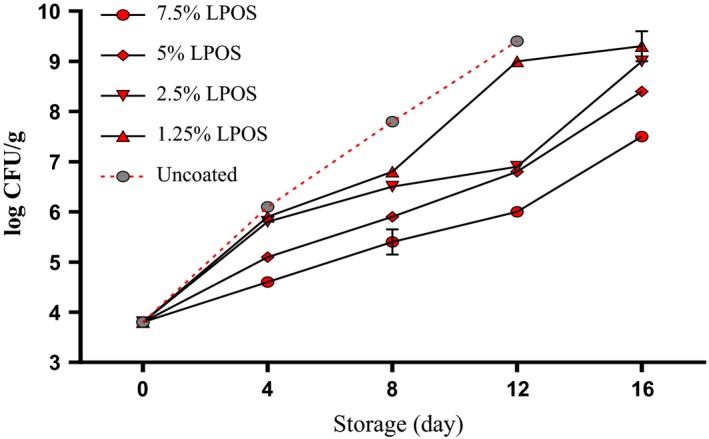
The *Pseudomonas* spp. changes of shrimp fillets as affected by LPOS‐WPS during refrigerated storage (4 ± 1°C). Values are the mean ± *SD*. LPOS, lactoperoxidase system; WPS, whey protein solution

The growth of *Pseudomonas* spp. in shrimp samples during the storage is shown in Figure 3. The *Pseudomonas* spp. as aerobic bacteria and *psychrotrophic* bacteria is affected by LPOS and whey protein coating solution. The *Pseudomonas* spp. counts in the control group during storage period were higher than groups of the LPOS‐treated. In counting these bacteria, by increasing the concentration of LPOS in coating solution of whey protein, fewer bacteria were observed although there was no significant statistical difference between two lower levels of LPOS (1.25% and 2.5%) up to the end of 12th day of storage. Furthermore, the biggest differences were between the level of 7.5% LPOS‐treated samples and control batch of *Pseudomonas* bacteria count; the differences in 4 and 12 days of storage were 1.34 and 2.75 log CFU/g, respectively. Therefore, LPOS in WPS showed a proper antibacterial feature on shrimp fillets against *mesophilic* and *psychrotrophic* bacteria.

**Figure 4:**
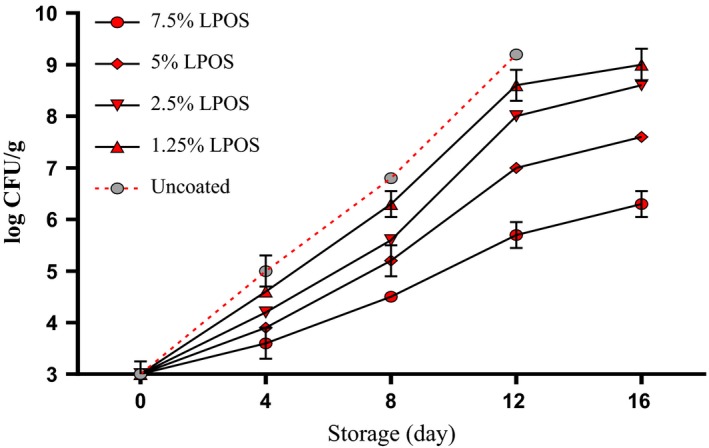
The *Pseudomonas fluorescens* changes of shrimp fillets as affected by LPOS‐WPS during refrigerated storage (4 ± 1°C). Values are the mean ± *SD*. LPOS, lactoperoxidase system; WPS, whey protein solution

**Figure 5:**
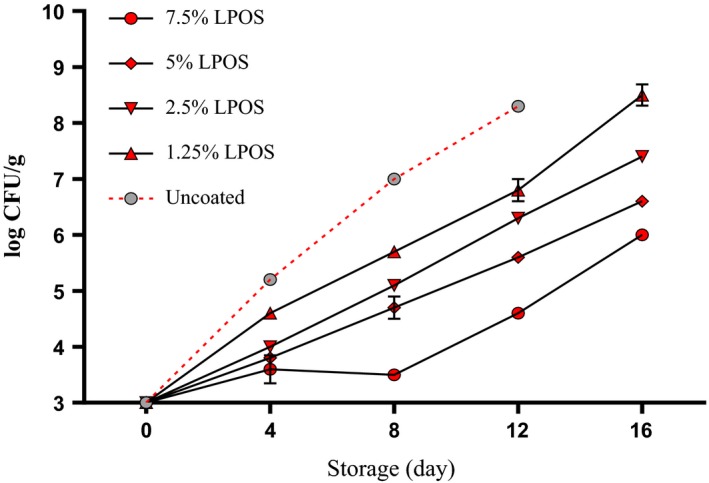
The *Shewanella putrefaciens* changes of shrimp fillets as affected by LPOS‐WPS during refrigerated storage (4 ± 1°C). Values are the mean ± *SD*. LPOS, lactoperoxidase system; WPS, whey protein solution

*Pseudomonas fluorescens* an obligatory aerobic bacterium and *Shewanella purefaciens* are another important bacterium as shrimp‐specific spoilage microorganism (Surendran et al., 1989; Xu et al., 2012). Finally, there was a significant difference (*p* < .05) in the counts of H_2_S producing bacteria (*P. fluorescens* (Figure 4) and *S. purefaciens* (Figure 5)) between the control group and LPOS‐treated groups especially in higher levels. At the end of the last day of storage (16th day), the number of these two bacteria was less than 7 log cfu/g for 7.5% LPOS‐treated fillets. It should be noted that statistically significant differences between the H_2_S‐producing microorganisms in the 7.5% LPOS with control group on 8th and 12th sampling days were more than 2 log CFU/g. So inhibiting the growth of spoilage bacteria was in the category containing the highest level of LPOS. The role of *P. fluorescens* and *S. putrefaciens* as specific spoilage bacteria in the degradation of seafood has been proven (Surendran et al., 1989; Xu et al., 2012). Therefore, the activation of the LPOS as bio‐preservative agent used in this study can slow the deterioration of shrimp quality. The LPOS can be used as a natural preservative, among other factors known to be effective in maintaining the quality of shrimp. The microbial analysis of results obtained in this study corresponded well with the results of other researches. The studies showed that in the presence of SCN^−^ and H_2_O_2_, lactoperoxidase enzyme produces substances such as OSCN^−^ and HOSCN, which are strong oxidizing agents with the oxidation of sulfhydryl groups in enzymes and proteins of bacteria (Kamau, Doores, & Pruitt, 1990). The results of this study corresponded with the results of the study by Shokri, Ehsani, & Jasour (2015) that reported the impact of the LPOS in whey protein to record the inhibition of aerobic bacteria assessment in rainbow trout during storage. This study also is consistent with the study by Yener et al. (2009) performed on the antimicrobial activity of LPOS with alginate coating films and found that growth of bacteria *Escherichia coli*,* Listeria innocua,* and *P. fluorescens* is delayed for a specific period of time at the presence of 0.4 or 0.8 mmol/L of H_2_O_2_ and 4 mmol/L of KSCN.

### 3.2 Chemical analysis

#### 3.2.1 Assessment of TVB‐N changes

**Figure 6:**
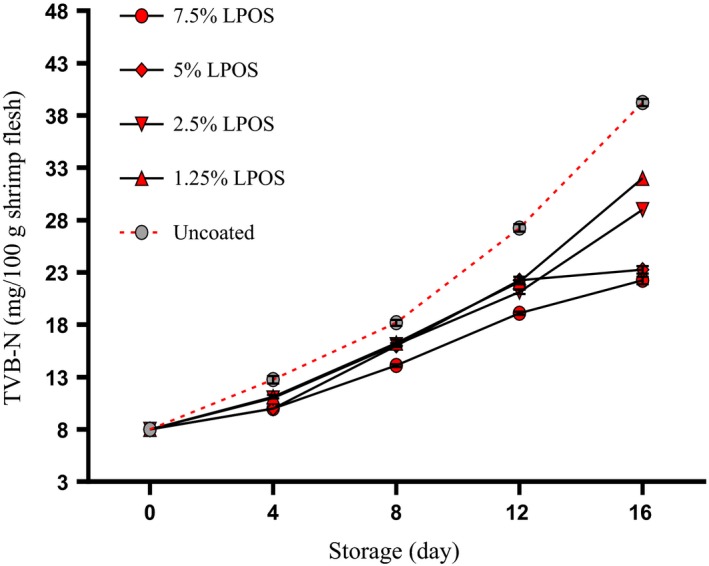
TVB‐N changes of shrimp fillets as affected by LPOS‐WPS during refrigerated storage (4 ± 1°C). Values are the mean ± *SD*. LPOS, lactoperoxidase system; WPS, whey protein solution

Crustacean meat is full of amino acids and the soluble nitrogen; therefore, the increased total volatile base nitrogen is one of the indicators of shrimp corruption. The transformation of trimethylamine oxide (TMAO) to trimethylamine (TMA) using enzymes in the microorganism is the reaction which results in the production of volatile base nitrogen (Lu, 2009). Changes of TVB‐N values for shrimp fillets are shown in Figure 6. In this study, at the start of the experiment, the TVB‐N value of the samples was 8–8.5 mg N/100 g meat. This result is consistent with the results of other studies which were 25 mg N/100 g meat (Ojagh, Rezaei, Razavi, & Hosseini, 2010). The TVB‐N during storage period increased (*p* < .05); so, there was a good correlation between the storage time and the TVB‐N formation (*r* = .96–.99). The LPOS combined with a solution of whey protein led to inhibition of the formation of TVB‐N compared to the control group at all sampling times. So the LPOS is useful in decreasing the production of the TVB‐N by microorganisms. The reason could be the antimicrobial activity of the LPOS. The researchers have demonstrated that the high acceptability limit value for seafood was 30–35 mg TVB‐N/100 g flesh (Huss, 1988; Kykkidou, Giatrakou, Papavergou, Kontominas, & Savvaidis, 2009). In the present study, the TVB‐N for the control group on 16th day was more than the limit of acceptability, right at the same time this group was also unacceptable considering sensory properties. TVB‐N values for the 1.25%, 2.5%, 5%, and 7.5% LPOS‐treated shrimps even after 16 days were still much less than being unacceptable. When the unacceptability of control group was declared by the panel list, TVB‐N values were 39.50 mg/100 g for control group. Our observation was in agreement with Yousefi et al. (2018) reported that the effects of LPOS‐alginate coating can prevent the formation of TVB‐N in chicken breast fillets during cold storage. Similar results were reported by Shokri, Ehsani, and Jasour (2015), as well. These results suggest that the measurement of TVB‐N could be an indicator used to evaluate the spoilage of shrimp stored at 4°C.

#### 3.2.2 Assessment of TBA changes

**Figure 7:**
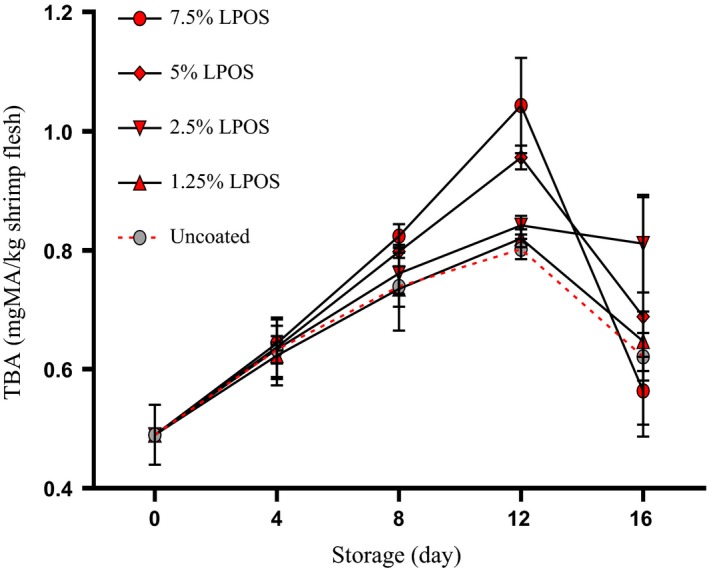
TBA changes of shrimp fillets as affected by LPOS‐WPS during refrigerated storage (4 ± 1°C). Values are the mean ± *SD*. LPOS, lactoperoxidase system; TBA, thiobarbituric acid; WPS, whey protein solution

Thiobarbituric acid test measured the malondialdehyde (MDA) content that can be used to determine the rate of lipid oxidation. MDA is one of the secondary products of oxidation produced by degradation of lipid hydroperoxides (Bensid, Ucar, Bendeddouche, & Özogul, 2014). Kilinc, Cakli, Dincer, and Tolasa (2009) determined the TBA value in high‐quality materials to be less than 3 mg MDA/kg, also the value for good quality food should not be more than 5 mg MDA/kg. As shown in Figure 7, in this study, TBA value at the start of storage period for shrimp samples was found to be 0.49 mg MDA/kg. TBA values in all group samples increased at first and by time‐lapse, this amount decreased or had no significant changes. The initial increase in TBA value is related to the formation of secondary lipid oxidation compounds, while reducing the TBA values can be related to the breaking of the formation of MDA because of tertiary degradation (Ehsani, Jasour, Hashemi, Mehryar, & Khodayari, 2014). On all days of testing storage, more TBA value was observed for the LPOS‐treated samples compared with the control group. However, there was no meaningful differences (*p* > .05) between any of the treatments. Thus, it can be concluded that LPOS activation in coating solutions of whey protein could not be effective in the TBA value although TBA formation was observed in a slow rate. These findings are in agreement with those of Shokri, Ehsani, & Jasour (2015), who reported that TBA values for Rainbow Trout Fillets did not meaningfully differ between control and LPOS‐treated samples. TBA values of all groups, except control group, got to its highest amount on the 12th day of storage, while panel members did not recognize any odor during these 12 days. The similar results were observed by Rostami et al. (2017) and Jasour et al. (2015). Rostami et al. (2017) reported that LPOS incorporated with whey protein coating cannot have any inhibitory effect on the lipid oxidation in Pike‐Perch fillets during cold storage. According to these results, it is difficult to determine limits for TBA test. It does not seem that TBA value could be an appropriate index for measuring the actual amount of lipid oxidation, as MDA can react with other components of shrimp, such as proteins, amino acids, nucleic acids, nucleosides, and phospholipids (Bensid et al., 2014; Aubourg, 1993), and produce secondary compounds, including, furfural, carbohydrates, alkadienals, alkenals and other aldehydes, and ketones (Bensid et al., 2014; Botsoglou et al., 1994).

#### 3.2.3 Determination of pH changes

**Figure 8:**
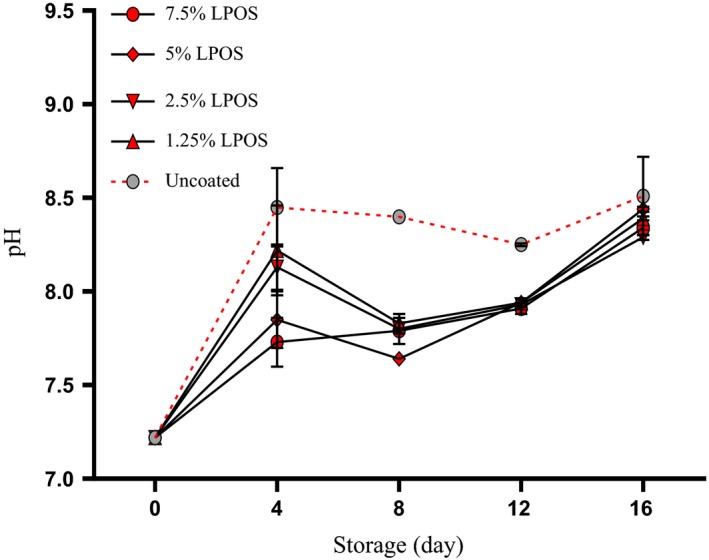
pH changes of shrimp fillets as affected by LPOS‐WPS during refrigerated storage (4 ± 1°C). Values are the mean ± *SD*. LPOS, lactoperoxidase system; WPS, whey protein solution

Figure 8 shows the changes in pH value for shrimp fillet during storage period at refrigerator temperature. The pH of the shrimps at the start of storage was 7.22. The pH values of shrimp fillets studied during storage increased and decreased respectively and increased again. However, for the control group, pH has risen significantly during the storage to achieve a value of 8.51 till the end of storage. The increase in the pH of the shrimp was owning to the gathering of alkaline compounds like ammonia compounds and trimethylamine due to activity of enzymes or bacteria (López‐Caballero, Martínez‐Alvarez, Gómez‐Guillén, & Montero, 2007). According to another examination done during the same time of keeping in the refrigerator, measuring the pH of the samples for 5% and 7.5% of LPOS‐treated samples has shown lower pH values than other groups (*p* < .05). Thus, the differences in pH at the end of the storage time was more than 0.50 pH units, showing that the incorporation of LPOS to the coating solution decreased the bacterial and endogenous alkalinizing mechanisms which may limit the shelf life of shrimp. Similar findings were reported by Yousefi et al. (2018), Rostami et al. (2017), Jasour et al. (2015), and Shokri, Ehsani, & Jasour (2015). According to the results obtained, the pH values of all samples were less than the range of acceptability even at the sensory rejection time; this could be resulted in that pH was not suitable quality index.

### 3.3 Determination of sensory score changes

As shown in Table 1, by the time‐lapse, all of the experimental groups decreased in terms of sensory attributes acceptability. For the LPOS‐treated batches by activating LPOS‐WPS, the best results of sensory attributes were achieved. In the assessment, the control group received less score in comparison with other groups. WPS of LPOS‐treated samples, particularly at high concentration (5%, 7.5%), had high acceptability in most of the organoleptic characteristics studied (*p* < .05). So that even at the end of the 16 days of storage in the refrigerator, it was still acceptable in qualitative terms for members of the panel. The result confirms LPOS as a preservative in using besides coating solution of whey protein. The control group and (1.25%) LPOS‐treated samples have shown the highest level of corruption in the refrigerator during the storage, respectively; and up to 12th and 16th days of storage of the acceptance, they were of acceptable quality. It seems that this acceptance was owing to the use of LPOS in the samples. However, the levels of 5% and 7.5% LPOS at the end of the storage have had high acceptability. The microbiological results obtained in this study were confirmed declining rate of microbial growth. Hence, the levels of 5% and 7.5% LPOS‐treated shrimps were chosen as the best levels of sensory and microbiological quality. Our observations are in accordance with Jasour et al. (2015) and Shokri, Ehsani, & Jasour (2015) reported that incorporated LPOS with chitosan and whey protein coating, respectively, could increase the shelf life of fish fillets during 16 days in cold storage in terms of sensory evaluation.

**Table 1:** Sensory score changes of shrimp fillets as affected by LPOS‐WPS during refrigerated storage (4 ± 1°C)

Sensory parameter	Treatment	Storage time (days)				
		0	4	8	12	16
Odor	Control	8.70 ± 0.27^aD^	7.41 ± 26^aC^	5.47 ± 0.32^aB^	3.43 ± 0.29^aA^	NE
	1.25%LPOS	8.73 ± 0.25^aE^	7.62 ± 0.32^aD^	6.59 ± 0.27^bC^	5.19 ± 0.27^bB^	3.24 ± 0.25^aA^
	2.5%LPOS	8.71 ± 0.32^Ad^	8.44 ± .019^bD^	7.17 ± 0.35^cC^	5.95 ± 0.21^cB^	4.74 ± 0.31^bA^
	5%LPOS	8.75 ± 0.21^aD^	8.42 ± 0.29^bD^	7.40 ± 0.23^cB^	6.51 ± 0.33 ^dB^	5.63 ± 0.22^cA^
	7.5%LPOS	8.73 ± .026^aC^	8.68 ± 0.14^bC^	8.27 ± 0.26 ^dB^	7.41 ± 0.0.24^eA^	6.97 ± 0.29^dA^
Color	Control	8.90 ± 0.19^aD^	8.39 ± 0.18^aC^	6.28 ± 0.14^aB^	4.91 ± 0.33^aA^	NE
	1.25%LPOS	8.83 ± 0.26^aD^	8.64 ± 0.17^aD^	7.32 ± 0.27^bC^	5.40 ± 0.23^aB^	3.92 ± 0.22^aA^
	2.5%LPOS	8.76 ± 0.13^aC^	8.38 ± 0.22^aC^	7.36 ± 0.39^bB^	6.03 ± .024^bA^	5.61 ± 0.19^bA^
	5%LPOS	8.80 ± 0.27^aE^	8.30 ± 0.39^aD^	7.52 ± 0.28^bC^	6.90 ± 0.33^cB^	6.10 ± 0.27^cA^
	7.5%LPOS	8.77 ± 0.20^aD^	8.29 ± 0.34^aC^	7.63 ± 0.32^bB^	7.29 ± 0.26^cAB^	6.91 ± 0.23^dA^
Texture	Control	8.94 ± 0.13^aD^	8.55 ± 0.14^aC^	4.63 ± 0.35^aB^	3.34 ± 0.22^aA^	NE
	1.25%LPOS	8.94 ± 0.14^aC^	8.61 ± 0.21^aC^	6.42 ± 0.21^aB^	5.49 ± 0.21^aA^	5.14 ± 0.27^aA^
	2.5%LPOS	8.90 ± 0.16^aD^	8.71 ± 0.18^aD^	7.84 ± 0.29^aC^	6.80 ± 0.28^aB^	5.63 ± 0.23^bA^
	5%LPOS	8.89 ± 0.21^aC^	8.76 ± 0.25^aC^	8.48 ± 0.26^bC^	7.66 ± 0.33^bB^	7.07 ± 0.16^cA^
	7.5%LPOS	8.93 ± 0.25^aC^	8.83 ± 0.24^aC^	8.36 ± 0.29^bBC^	7.91 ± 0.29^bB^	7.28 ± 0.29^cA^
Overall acceptability	Control	8.93 ± 0.21^aD^	7.61 ± 0.28^aC^	5.35 ± 0.33^aB^	3.62 ± 0.23^aA^	NE
	1.25%LPOS	8.86 ± 0.33^aE^	8.13 ± 0.27^bD^	6.58 ± 0.32^bC^	5.28 ± 0.32^bB^	3.75 ± 0.17^aA^
	2.5%LPOS	8.83 ± 0.17^aD^	8.43 ± 0.34^bD^	7.34 ± 0.26^cC^	6.07 ± 0.27^cB^	5.40 ± 0.21^bA^
	5%LPOS	8.85 ± 0.21^aD^	8.55 ± 0.17^bCD^	8.10 ± 0.17^dC^	6.94 ± 0.21 ^dB^	6.25 ± 0.26^cA^
	7.5%LPOS	8.83 ± 0.26^aD^	8.63 ± 0.31^bCD^	8.31 ± 0.13^dBC^	7.89 ± 0.36^eAB^	7.49 ± 0.27^dA^

Different lower case superindex letters within a column are significantly different (*p* < .05). Scores are given as mean ± *SD*.

Scale from 9 to 0 (limits of unacceptability, moderate acceptability, and high acceptability indicates the scores 1–3.9, 4–6.9, and 7–9, respectively.)

LPOS, lactoperoxidase system; NE, not evaluated; WPS, whey protein solution.

## 4 CONCLUSIONS

The combination of the LPOS with whey protein coating has been protected significantly against some bacterial growth and TVB‐N formation, while it did not have significant effect on the rate of lipid oxidation compared with the control group at all sampling time. The results of sensory evaluation showed the effectiveness of the LPOS use in maintaining the quality and increasing the shelf life of shrimp fillets kept in the refrigerator. It is noteworthy that in sensory evaluation, as well as microbial test and measuring TVB‐N, by increasing the concentration of LPOS in the whey protein coating, better results were observed in terms of proximity to limits of acceptable measurement indexes for shrimp meat. Accordingly, not only with microbial data but also with sensory evaluation, the shelf life of the shrimp fillet coated with LPOS and WPS was recorded for 12 days in the refrigerator. While using 1.25% (v/w) LPOS compared to the control, sample was increased the shelf life of 4 days. The shelf life of shrimp fillets when using 2.5%, 5%, and 7.5% LPOS indicated a medium or high general acceptability up to the 16th day of storage. Thus, the samples treated with 5% and 7.5% LPOS were chosen as the best samples in the sensory and bacterial analysis. Generally, LPOS use, with whey protein coating, can extend shelf life of shrimp fillet during storage in the refrigerator.

## 5 FUTURE TRENDS

- Investigating the effects of LPOS with antioxidant substances such as essential oils and extracts in food.
- Study of the effects of LPOS with other edible coatings and films.
- Investigating the effects of LPOS on other foods.
- Investigating the effect of LPOS on the shelf life of perishable food products against fungi, such as some fruits, due to the antifungal activity of this system.
- Investigating the effect of LPOS on the virus transmitted through food.

## CONFLICT OF INTEREST

The authors declare that there is no conflict of interests.

## ETHICAL REVIEW

This article does not involve any studies with human or animal subjects.
